# Family studies in Gaucher Disease: a key resource for early diagnosis and personalized treatment strategies

**DOI:** 10.1186/s13023-026-04371-w

**Published:** 2026-04-28

**Authors:** Martina Vinci, Miriam Giacomarra, Annalisa D’Errico, Rita Fischetto, Giovanna Palumbo, Immacolata Tartaglione, Paolo Tirelli, Elisa Messina, Maria Russo, Daniele Francofonte, Paolo Colomba, Giovanni Duro, Carmela Zizzo

**Affiliations:** 1 Institute for Biomedical Research and Innovation (IRIB), National Research Council (CNR), 90146 Palermo, Italy; 2 Clinical Genetics Unit, Department of Pediatric Medicine, XXIII Children’s Hospital, Bari, Italy; 3 Department of Translational and Precision Medicine, “La Sapienza” University of Rome, 00161 Rome, Italy; 4 Department of General and Specialized Surgery for Women and Children, University of Campania “Luigi Vanvitelli”, 80131 Naples, Italy; 5 Department of Internal Medicine, “Ospedale del Mare” Hospital, 80147 Naples, Italy

**Keywords:** Gaucher Disease, Family segregation studies, Genetic analysis, Personalized medicine

## Abstract

Gaucher Disease (GD) is an inherited metabolic disorder caused by mutations in the *GBA1* gene, which is responsible for the synthesis of the enzyme glucocerebrosidase *(GCase).* The clinical manifestations, which are extremely heterogeneous, include splenomegaly, hepatomegaly, anaemia and bone complications. GD is an autosomal recessive condition, meaning that the clinical phenotype manifests itself only in the presence of two mutated alleles, which are inherited from both parents, who are generally healthy and asymptomatic. However, other family members (brothers, sisters, grandparents, uncles, aunts, cousins) may be heterozygous carriers or, in some cases, present with undiagnosed forms. In order to identify these individuals and monitor their health, it is essential to conduct family segregation studies. These are often disregarded in clinical practice, despite their crucial role in understanding the distribution of the disease. In this study, a comprehensive diagnostic analysis was conducted on four families, including biochemical and genetic investigations. In the first three families, an affected proband was identified, exhibiting characteristic symptoms, reduced enzyme activity and increased substrate, associated with the presence of two causative mutations. Segregation studies revealed additional affected individuals or carriers, some of whom were completely asymptomatic or had mild manifestations. In the fourth family, the investigation started with a paternal uncle who was found to be a heterozygous carrier. The study was extended to non-direct relatives, which allowed the identification of other affected individuals who would otherwise have remained undiagnosed. These results emphasise the significance of extended family analysis, encompassing second-and third-degree relatives, as a fundamental instrument for comprehension of the hereditary distribution of GD and for the timely identification of individuals at risk. The exclusion of non-direct relatives from genetic screening represents a significant missed opportunity for timely diagnosis, effective clinical management, and family planning.

## Introduction

 GD (OMIM #230800, ORPHA355) is a rare metabolic disorder caused by mutations in the *GBA1* gene, located on chromosome 1 (1q21), which encodes the lysosomal enzyme GCase, (EC: 4.2.1.25) [1]. Within lysosomes, this enzyme plays a crucial role in the hydrolysis of a lipid substrate, glucosylceramide (GlcCer), into ceramide and glucose. Deficiency of GCase activity leads to accumulation of its substrate in the lysosomes of macrophages, resulting in enlargement and subsequent designation as “Gaucher Cells” [2, 3]. These cells can infiltrate the bone marrow, liver, spleen and other organs, causing the characteristic symptoms of the disease [4].

Depending on the presence and severity of the primary central nervous system (CNS) disease, GD manifests in three main phenotypes: type 1 (or non-neuropathic OMIM #230800), type 2 with acute neuronal involvement (OMIM #230900) and type 3 with subacute neuronal evolution (OMIM #231000) [5, 6]. Type 1 is the most common form of the disease and generally does not involve neurological damage. It is characterized by a combination of hepatosplenomegaly, skeletal abnormalities (such as osteopenia, bone lesions and osteonecrosis) and haematological complications [7]. Type 2, in opposite, is characterized by the presence of neurological damage, with onset in the first years of life (before the age of two), manifesting as delayed psychomotor development and a rapid course leading to death within two or four years of life [8, 9]. Despite presenting with neuronal impairment and an early onset of symptoms, type 3 has a more gradual course, enabling patients to survive into their third or fourth decade of life [10]. However, it is important to note that this classification is not definitive because GD can present very differently in different individuals [2]. In fact, diagnosing the disease can be extremely difficult as the symptoms vary and often overlap with those of other conditions. For instance, an enlarged liver and spleen may be misinterpreted as a sign of other metabolic or infectious diseases, and haematological changes may not immediately suggest a rare condition such as GD [11]. This can lead to significant diagnostic delays and serious complications in patients who could have benefited from early treatment with enzyme replacement therapy (ERT) or substrate reduction therapy (SRT) [12].

GD is often diagnosed several years after the first clinical and laboratory signs appear. This is a common issue with rare diseases, which are characterized by a gradual onset of symptoms [4]. Diagnostic suspicion of GD is initially based on clinical signs and family history, and is subsequently confirmed by biochemical tests. These include measuring *GCase* enzyme activity on a dried blood spot (DBS), which is considered the first-level test. The activity may be zero or reduced compared to the reference value [13, 14]. Furthermore, the identification of specific GD biomarkers represents a significant advance in diagnosis, as it enables more precise evaluation of disease severity and the efficacy of adopted therapies, thereby enhancing overall diagnostic accuracy [15]. Among these, Glucosyl Sphingosine (LysoGb1) is the biomarker most commonly used in the diagnosis of GD. It enables early detection of the disease, even in the absence of overt clinical signs. Its measurement in blood or urine is highly sensitive and specific. It also supports disease subtyping and monitoring of treatment response [16–18].

Finally, Lyso-Gb1 measurement in DBS represents a promising option for neonatal screening. It provides a non-invasive and practical approach for early identification of the disease. It also allows more accurate patient follow-up [17, 19].

**Fig. 1:**
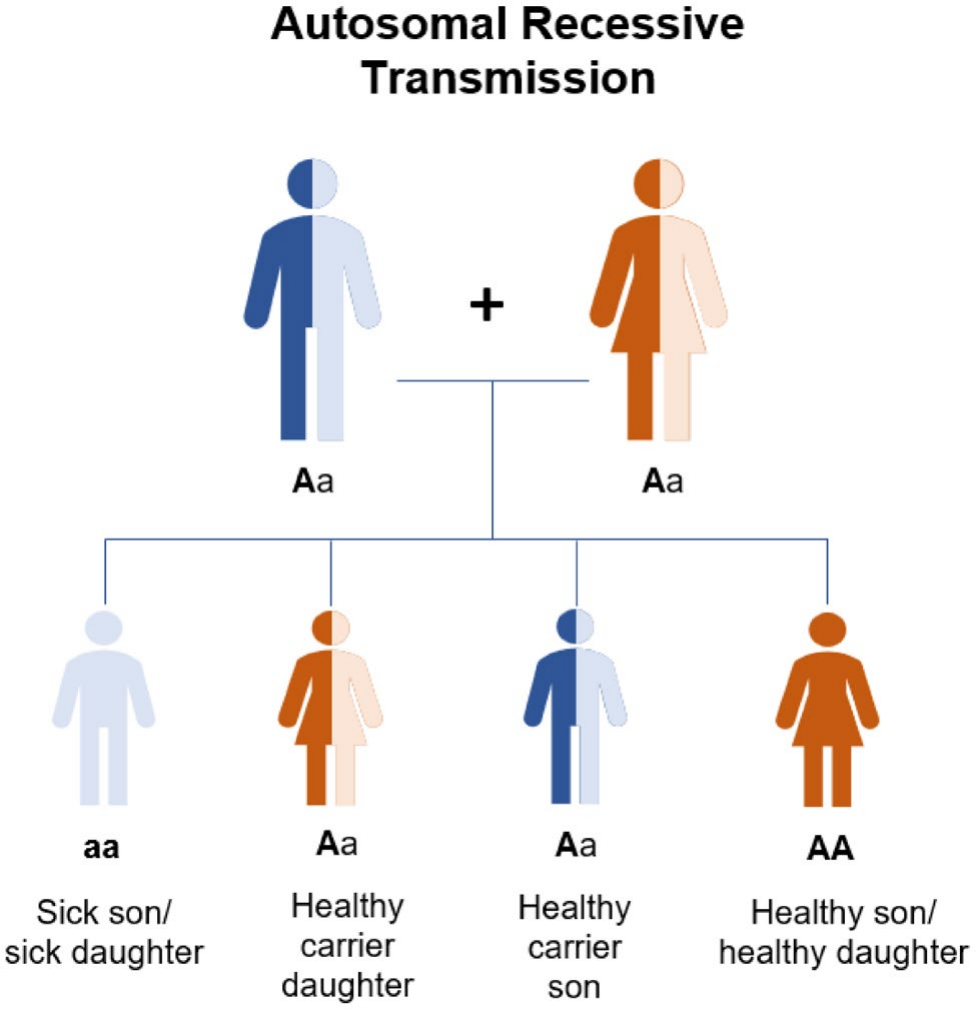
Autosomal recessive transmission pattern in GD. Male subjects are shown in blue and female subjects in orange. A indicates the healthy gene, while a indicates the mutated gene. Parents are heterozygous carriers (Aa); children may be healthy carriers (Aa 50%), healthy (AA 25%) or affected (aa 25%)

Although enzymatic analysis is considered the main discriminating tool in GD, genetic analysis is still important for clarifying specific genetic variants, supporting genetic counselling and helping to establish the severity of the disease. The pathology is characterized by an autosomal recessive pattern of transmission, which implies that the disease manifests only when an individual inherits two mutant copies of the *GBA1* gene, one from each parent [3]. Specifically, in a couple where both parents are healthy carriers (heterozygous), each child has a 25% chance of inheriting two normal copies of the gene (healthy individual), a 50% chance of being a healthy carrier (one mutated and one normal copy), and finally a 25% chance of inheriting two mutant copies of the gene, resulting in the disease [20] (Fig. 1).

**Fig. 2:**
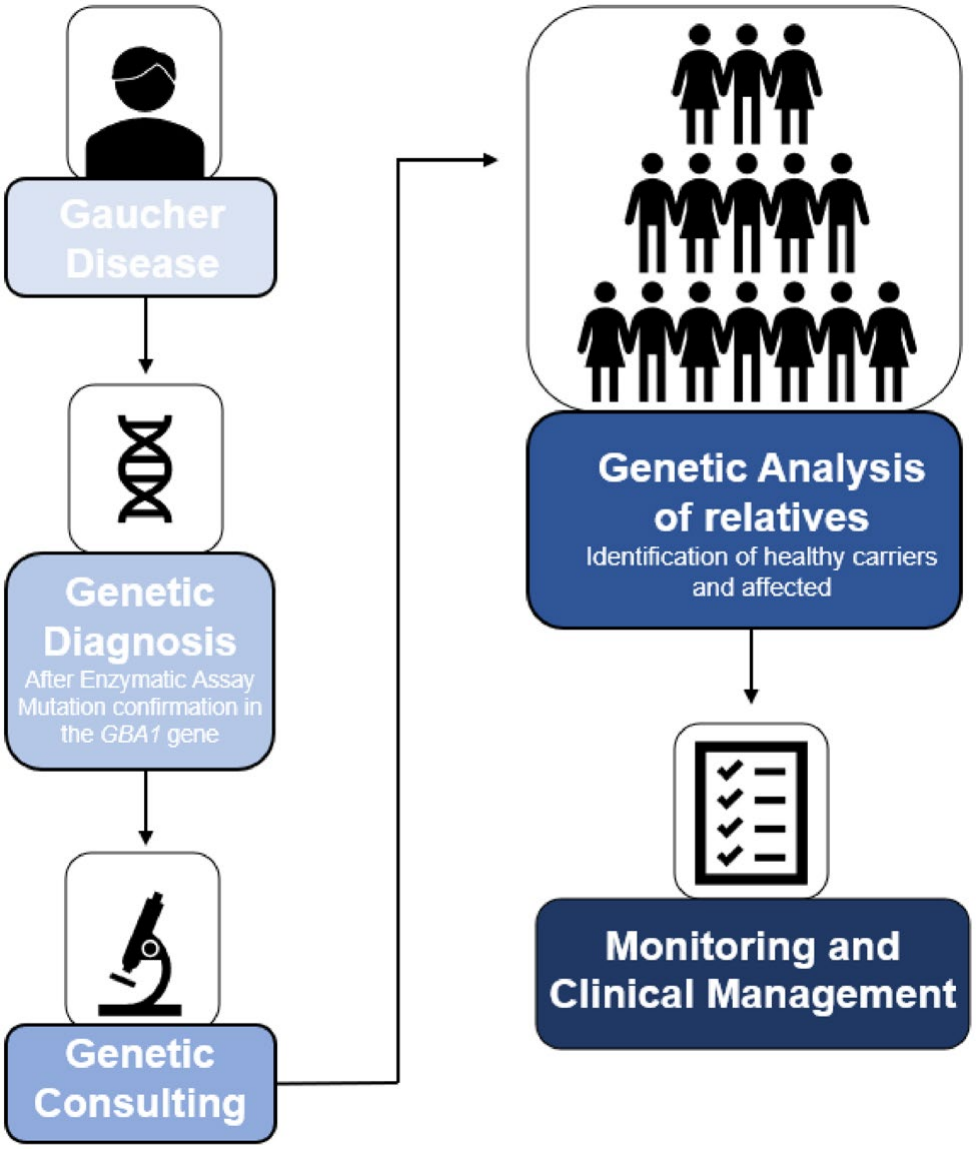
Flow chart for a patient with GD. Following the confirmation of the mutation in the GBA1 gene, genetic counselling and family segregation studies are conducted, with the subsequent identification of affected family members and healthy carriers. Consequently, the monitoring and clinical management of the disease are initiated

Typically, healthy carriers do not manifest any clinical symptoms. In this context, family studies are crucial for early diagnosis and proper disease management. They enable the identification of healthy carriers, as well as the early detection of affected individuals and those at risk. Indeed, in-depth family history and targeted genetic testing allow asymptomatic cases to be identified and early intervention to be carried out, reducing the risk of serious complications and improving the efficacy of treatment *(*Fig. 2*).* However, despite the numerous benefits of family genetic mapping, this investigation is sometimes underestimated in clinical practice. Clinicians may not adequately consider the importance of genetic evaluation in apparently asymptomatic patients or in families without a clear history of disease, thus limiting the possibilities for early intervention and optimal management of the disease.

In this study, four distinct families were analyzed: in the first three, a proband affected by GD was identified, while in the fourth family, genetic testing was initiated not from a direct relative, but from the paternal uncle, who was found to be a heterozygous carrier of a pathogenic variant in the *GBA1* gene. Subsequent family segregation analysis then enabled the identification of additional affected members or heterozygous carriers, including individuals from previous generations, such as grandparents, uncles, and nephews. The four cases were selected on the basis of clinical symptoms suggestive of GD. Biological samples (DBS) were sent to our laboratory by clinicians from various specialist centres in Italy. Following diagnostic confirmation, the study was extended to family members in order to carry out genetic segregation analysis, in line with our study design. The present work aims to emphasise the strategic value of family studies in the field of autosomal recessive diseases, such as GD. Although often underestimated in clinical practice, these investigations are essential for identifying individuals in the pre-symptomatic phase. This allows early access to specific therapies and may help prevent irreversible organ damage. Moreover, family studies facilitate the identification of carriers of pathogenic variants in the *GBA1* gene, a condition that is now recognized as an increased risk factor for the development of Parkinson’s disease [21, 22].

## Materials and methods

### Patients

Peripheral blood was collected using ethylenediaminetetraacetic acid (EDTA) as an anticoagulant, following which it was dried on absorbent paper (dried blood spot, DBS). Genetic and enzymatic studies were performed at the Centre for Research and Diagnosis of Lysosomal Storage Disorders of IRIB-CNR in Palermo and were approved by the Hospital Ethics Committee of the University of Palermo. Signed informed consent was obtained from patients.

### Enzyme activity assays

The enzymatic activity of glucocerebrosidase was calculated using a fluorometric test on DBS, described by Chamoles et al. [23], with some modifications [unpublished data]. In summary, a spot containing 10 µL of blood in a 6 mm diameter paper circle was placed in a 96-well plate and, after an incubation period of 18 h at 37 °C (900 RPM), the reaction was stopped with 250 µL of 0.1 mol/L ethylenediamine (pH 11.4). The artificial substrate 4-methylumbelliferyl β-D-glucopyranoside (SIGMA-11.6 mmol/L) in phosphate-citrate buffer (CPB, pH 5.2), specific for *GCase*, conjugated with a fluorophore, was used. The fluorescence values detected by the fluorimeter (λex: 365 nm, λem: 448 nm) reflect the amount of substrate degraded by the enzyme present in the analysed sample. The fluorescence data were processed by an algorithm that provided a value of enzymatic activity expressed in nmol/h/mL for each sample. In addition, we added a cell lysis solution at pH 5.2 (CPB, TRITON-X 100, SODIUM TAUROCOLATE) and CBE (Conduritol B epoxide), a specific inhibitor of non-lysosomal β-glucosidase (NLGCase), to the reaction mixture to discriminate between *GCase* and *NLGCase*. The values considered normal for GD were as follows: ≥ 2.5 nmol/L/h.

### DNA extraction

Genomic DNA was isolated from dried blood spots using silica-coated magnetic particles in an automated nucleic acid purification extractor (EZ1&2 DNA Investigator Kit, Qiagen, Hilden, Germany). DNA concentration was measured using a biophotometer (D30, Eppendorf, Hamburg, Germany).

### PCR and sanger sequencing

The search for mutations in the *GBA1* gene was performed using Sanger sequencing. Two pairs of PCR primers were designed to analyse *GBA1* exclusively and identify recombinant alleles with *GBAP* at a concentration of 0.4 µM in the reaction mixture. Long-run PCR amplification of the two macroregions, exon 1–intron 5 and intron 5–exon 11 (two reaction mixtures for each sample), was performed using biotechrabbit™ Long-Run PCR Master Mix (Voden Medical Life Science and Diagnostic Division, Meda, MB, Italy), 2X, following the basic protocol and cycling program described in the reaction manual. PCR Enhancer and MgCl₂ were not included in the mixture. The template DNA concentration was 50–100 ng in each reaction mixture. The exon 1–intron 5 and intron 5–exon 11 PCR products were purified using ExoSAP-IT™ PCR and used for sequencing of exons 1–5 and 6–11 of *GBA1*. Sanger sequencing was performed by Eurofins Genomics (Vimodorne, MI, Italy). Sequence analysis was performed using LI-COR Align IR (Lincoln, NE, USA) and Chromas 2.5 bioinformatics programs. Sequencing primers are listed in Table 1.

**Table 1:** PCR and sequencing primers for genetic analysis of *GBA1*. Nucleotide sequences of the primers used to carry out the genetic study are shown

GBA1 PCR Primers exon 1–intron 5	5′-3′ sequence
GBA_EX1FOR-PC	CTCCATGCAAATCTGTGTTC
GBA_EX5Rev-PC	GGCCTGAAAAAGCTAGAATG
GBA1 PCR Primers intron 5–exon 11	5′-3′ sequence
GBA_EX5FOR-PC	CCAGGATGATTGCGAACTC
GBA_EX11Rev-PC	TGCTGTGCCCTCTTTAGTC
GBA1 Sequencing Primers exon 1–intron 5	5′-3′ sequence
Ex1F seq GBA	AGATGAGAGGAAGCCAA
ex2R seq GBA	TGGTCTCAGTCACTCAAAAG
EX3F seq GBA	TCTTTTGAAACAGAGTCTT
EX4R_GBA	CAGAATGGGCAGAGTGAGAT
EX5F seq GBA	GGCCTCCCAAAGTGCTGG
GBA1 Sequencing Primers intron 5–exon 11	5′-3′ sequence
Ex6R seq bb	ATTGAGAGGCCCAAGGCT
Ex7R seq bb	CCCTAGAAAGGTTTCAAGCGA
EX8F_GBA	TCCAGGATCAGTTGCTCTTC
EX8R seq bb	AGTAAGAGGTCTGAGGTCTG
EX9F seq bb	TCTTACTAGTTTCACCAAAG
EX9R seq bb	AAGTTACGCACCCAATTGGG
EX9F seq bb2	CCTTGCCCTGAACCCCGAA

### LysoGb1 assay

LysoGb1 was determined on dried blood spots using tandem mass spectrometry (LC-MS/MS) [24–28].

### MLPA analyses

We performed MLPA analysis on DNA samples to identify the main rearrangements of the *GBA1* gene, using the SALSA MLPA P338-B2 *GBA* kit, in accordance with the manufacturer’s instructions (MRC Holland).

## Results

**Fig. 3:**
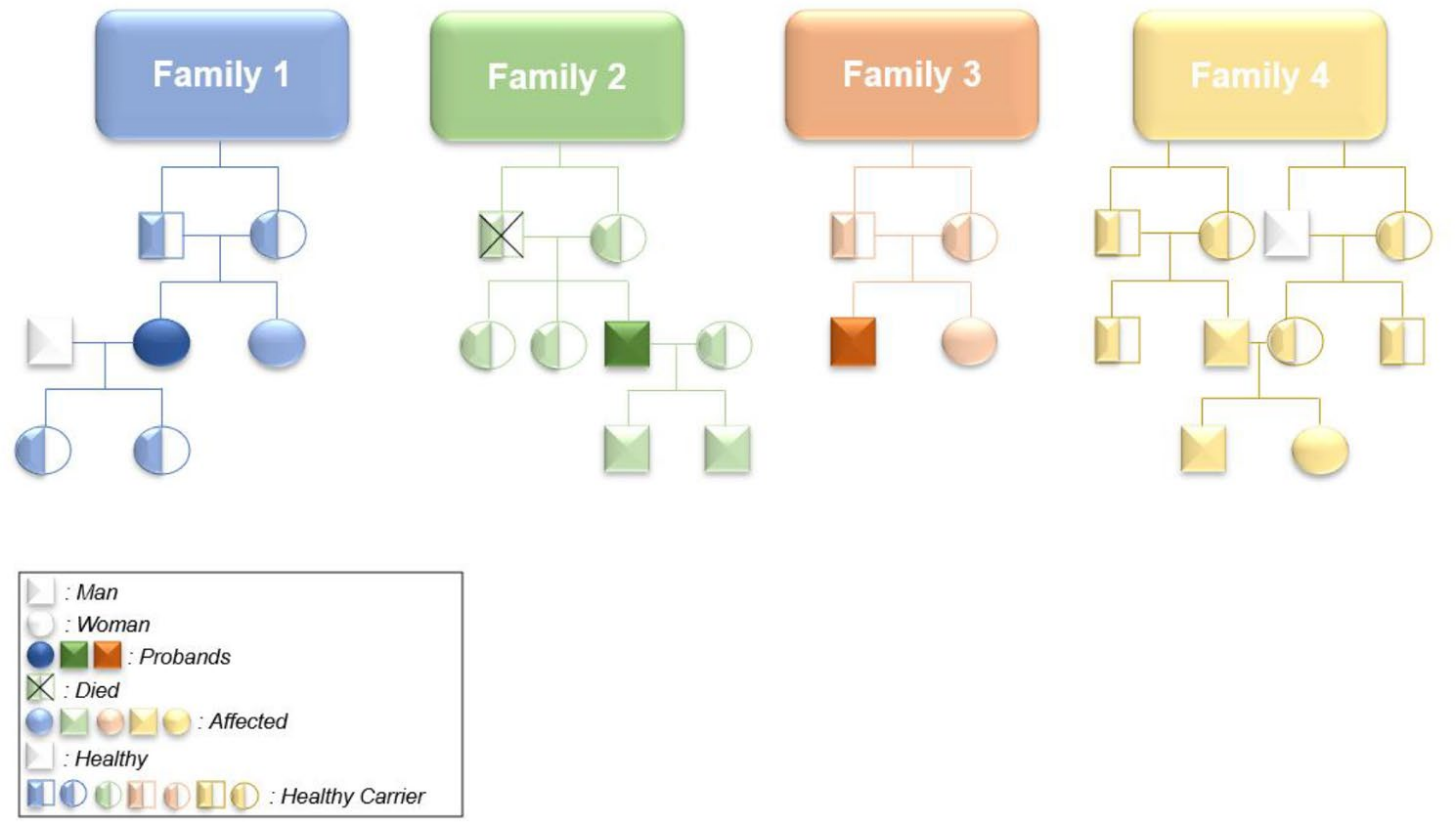
Graphical representation of the subjects studied for each family

In this study, four different families underwent a complete diagnostic examination for GD. In the initial three families, an affected proband was identified, exhibiting typical clinical symptoms, absent or reduced enzyme activity, accumulation of the LysoGb1 substrate, and the presence of two causative genetic mutations. The results obtained and the hereditary nature of the disease prompted us to also examine the relatives of the probands, many of whom were symptomatic but unaware of the family situation. Consequently, we were able to identify affected individuals with symptoms but without a previous diagnosis, as well as symptomatic and asymptomatic carriers. In family 4, the case proved to be of particular interest, as the GD study was initiated not by a direct relative but by the paternal uncle. This individual was found to be heterozygous for a pathogenic variant of the *GBA1* gene. Subsequent extended family analysis identified other affected members, including cases diagnosed in the neonatal period (Fig. 3).

### Clinical history family 1

The patient, a 48-year-old woman, was assessed in 2023 following the detection of anaemia and leucopenia during routine tests. Imaging studies (full abdominal ultrasound and CT scan) revealed heterogeneous hepatosplenomegaly with the presence of multiple angiomas. A subsequent bone muscle biopsy revealed a high proportion of macrophage elements with large cytoplasm, sometimes with a “wrinkled” appearance, suggesting GD. The subject also presented with osteopenia and polyclonal hypergammaglobulinaemia (Table 2).

Biochemical analysis carried out in our laboratories revealed a marked reduction in acid β-glucosidase activity (0.5 nmol/h/mL; reference values ≥ 2.5 nmol/h/mL) and a significant increase in LysoGb1 (244 ng/mL; reference values ≤ 6.8 ng/mL) (Table 2). Meanwhile, molecular analysis of the *GBA1* gene (eleven exons and flanking intronic regions) identified two compound heterozygous pathogenic variants in exon 9: c.1226 A > G (p.N409S, also known as N370S) [29] and c.1342G > C (p.D448H, also known as D409H) [30] (Table 2). The c.1226 A > G variant causes an Asn→Ser substitution at position 409 (370 after removal of the signal peptide), whilst the c.1342G > C variant causes an Asp→His substitution at position 448 (409 after removal of the signal peptide). Both are pathogenic in homozygous or compound heterozygous forms according to the literature and databases dedicated to GD [31–33].

A family segregation analysis was therefore performed. The parents (both aged 76) had enzyme activity within the normal range (6.4 and 5.5 nmol/h/mL) and normal LysoGb1 levels (4.6 and 6 ng/mL) (Table 2). Genetic analysis revealed the N370S variant in heterozygous form in the father and the D409H variant in heterozygous form in the mother, confirming their status as healthy carriers.

The proband’s sister (aged 45), with a history of occasional epigastric pain, showed reduced enzyme activity (1.3 nmol/h/mL) and elevated LysoGb1 (82.1 ng/mL) (Table 2). Genetic analysis identified the same two compound heterozygous variants (N370S and D409H), confirming the diagnosis of GD. This would not have been succeed if the family segregation study had not been performed, even considering the carrier status of the parents and the disease of the sister. In the proband’s twin daughters (aged 15, asymptomatic), acid β-glucosidase activity was within the normal range (3.1 and 5.1 nmol/h/mL) and LysoGb1 levels were 4.9 and 15.2 ng/mL (Table 2). Genetic analysis revealed the N370S variant in a heterozygous form in one twin and the D409H variant in a heterozygous form in the other, consistent with healthy carrier status. The LysoGb1 value of 15.2 ng/mL observed in one of the twins is consistent with the heterozygous presence of the D409H variant. Analysis by Sanger sequencing and MLPA did not reveal any further pathogenic variants.

Currently, the proband and her sister are undergoing ERT.

**Table 2:** Clinical and molecular information on the proband of family 1 and his relatives. Values in bold are pathological

Patient	Sex	Age	Mutation	GCase Activity (nmol/mL/h) Normal Range > 2.5 nmol/mL/h	Lyso-Gb1 in Blood (ng/mL) Normal Range < 6.8 ng/mL	Clinical Signs
Proband	F	48	**N370S/D409H**	**0.5**	**244**	Anaemia, leukopenia, hepatomegaly, splenomegaly, osteopenia, polyclonal hypergammaglobulinemia
Father	M	76	N370S	6.4	**//**	Arterial hypertension
Mother	F	76	D409H	5.5	//	Asymptomatic
Sister	F	45	**N370S/D409H**	**1.3**	**82.1**	Epigastric pain
Daughter 1	F	15	N370S	3.1	5.1	Asymptomatic
Daughter 2	F	15	D409H	4.9	**15.2**	Asymptomatic

### Clinical history family 2

For this family, the diagnostic work-up for GD started with the proband: a 13-year-old boy whose clinical presentation was characterized by hepatosplenomegaly, anaemia, thrombocytopenia, and gastroesophageal reflux (Table 3).

Biochemical analysis revealed reduced acid β-glucosidase activity (1.3 nmol/h/mL) and a significant increase in LysoGb1 (184.0 ng/mL) (Table 3). Genetic analysis using Sanger Sequencing identified two compound heterozygous variants in the *GBA1* gene: the N370S mutation in exon 9 [29] and the RecNci I2 allelic conversion: c.[1448 T > C; 1483 G > C; 1497 G > C], resulting from recombination with the *GBAP* gene. The RecNci I2 conversion includes the following mutations: (1) c.1448T > C (p.L483P, also known as L444P) [29, 34, 35], located in exon 10, which causes the Leu→Pro substitution at position 483 (444 after removal of the signal peptide); (2) c.1483 G > C (p.A495P, also known as A456P) [36, 37], in exon 10, resulting in the Ala→Pro substitution at position 495 (456 after removal of the signal peptide); (3) c.1497G > C (p.V499V, also known as V460V) [36, 37], in exon 10, a synonymous variant that does not alter the amino acid (Val→Val) at position 499 (460 after removal of the signal peptide).

Following the diagnosis in the proband, a family segregation analysis was carried out. The father (aged 50) exhibited symptoms including bone fragility, hepatosplenomegaly, anaemia and lymphadenopathy (Table 3). Acid β-glucosidase activity was markedly reduced (0.9 nmol/h/mL) and LysoGb1 was elevated (14.7 ng/mL) (Table 3). Genetic analysis revealed a homozygous N370S mutation in exon 9 of the *GBA1* gene (Table 3), thus confirming the diagnosis of GD. In view of the father’s homozygous condition, the analysis was extended to the paternal grandparents. The grandmother (aged 89), who exhibited no clinical symptoms, was found to be a heterozygous carrier of the N370S variant, with an enzyme activity of 6.8 nmol/h/mL (Table 3). The grandfather, who passed away in 2020, had not undergone testing; however, in accordance with the autosomal recessive pattern of inheritance, it is probable that he was a heterozygous carrier. The proband’s mother (aged 48), who was clinically asymptomatic, exhibited enzymatic activity within the normal range (6.3 nmol/h/mL) and was found to be a heterozygous carrier of the RecNci I2 allelic conversion (Table 3). The proband’s brother (17 years old), who was also asymptomatic, showed reduced enzyme activity (2.1 nmol/h/mL) and elevated levels of LysoGb1 (64 ng/mL). Genetic analysis revealed the presence of the N370S mutation and the RecNci I2 allelic conversion in compound heterozygosity, thus confirming the diagnosis of GD (Table 3). However, it should be noted that, in the absence of a targeted family study, it would not have been possible to arrive at this identification. The father’s sisters (aged 58 and 61), who were both asymptomatic, were also analysed. In both cases, the standard value for normal enzyme activity (7.9 and 4 nmol/h/mL) and the presence of the N370S mutation in a heterozygous state were detected, thus confirming their carrier status (Table 3). At the present time, the proband, the father and the brother are undergoing ERT.

**Table 3:** Clinical and molecular information on the proband of family 2 and his relatives. Values in bold are pathological

Patient	Sex	Age	Mutation	GCase Activity (nmol/mL/h) Normal Range > 2.5 nmol/mL/h	Lyso-Gb1 in Blood (ng/mL) Normal Range < 6.8 ng/mL	Clinical Signs
Proband	M	13	**N370S/REC1**	**1.3**	**184**	Hepatosplenomegaly, anaemia, thrombocytopenia and gastroesophageal reflux
Father	M	50	**N370S/N370S**	**0.9**	**14.7**	Bone fragility, hepatosplenomegaly, anaemia and lymphadenopathy
Mother	F	48	REC1	6.3	//	Asymptomatic
Brother	M	17	**N370S/REC1**	**2.1**	**64**	Asymptomatic
Paternal Grandmother	F	89	N370S	6.8	//	Asymptomatic
Paternal aunt	F	58	N370S	7.9	//	Asymptomatic
Paternal aunt	F	61	N370S	4	//	Asymptomatic

### Clinical history family 3

In this third family, the proband, a 24-year-old man, presented with a clinical picture suggestive of GD, characterized by thrombocytopenia, hepatomegaly and splenomegaly (Table 4).

**Fig. 4:**
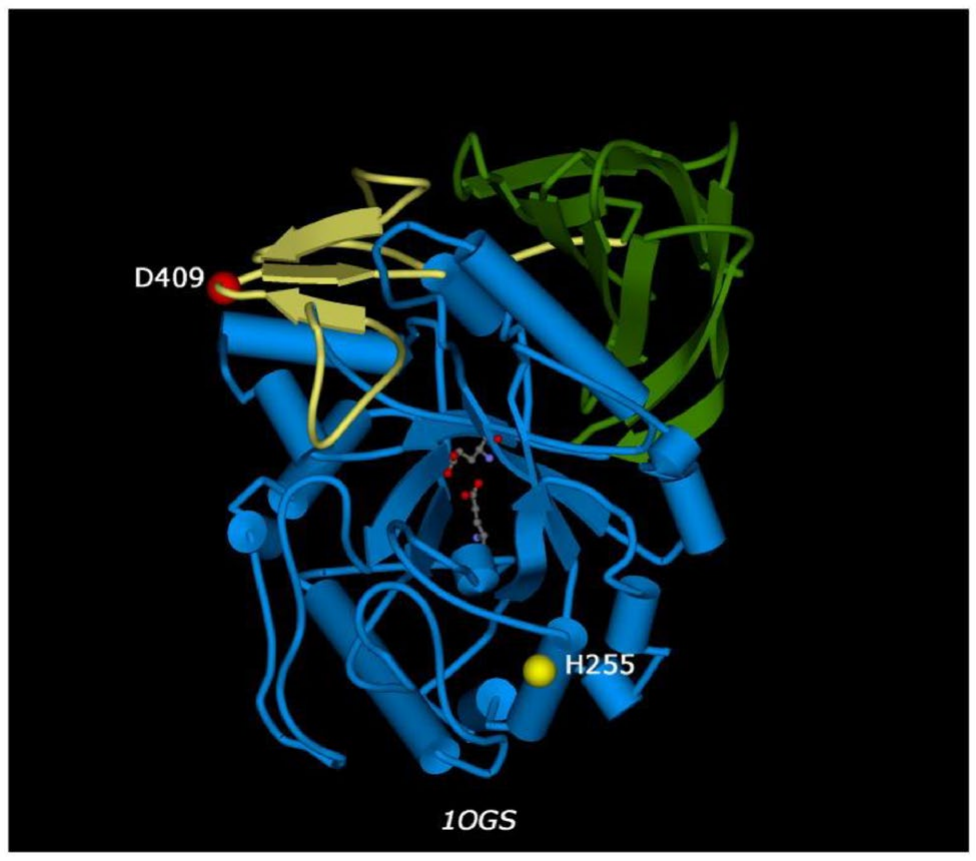
Threedimensional structure of human GCase, highlighting the two mutations D409H and H255Q on the same allele (https://pubmed.ncbi.nlm.nih.gov/19459886/) [39]

Biochemical analysis revealed a marked reduction in acid β-glucosidase activity (0.7 nmol/h/mL) and a significant increase in LysoGb1 (513 ng/mL) (Table 4). Molecular analysis of the *GBA1* gene, performed using Sanger Sequencing, identified a heterozygous mutational pattern consisting of the N370S variants and the complex allele H255Q+D409H (Table 4). In particular, the c.882 T > G variant in exon 7 results in the His→Gln substitution at position 294 (H294Q, also known as H255Q after removal of the signal peptide) [38]. The c.1342G > C variant in exon 9 results in the Asp→His substitution at position 448 (D448H, also known as D409H) [30]. The c.1226 A > G variant in exon 9 results in the Asn→Ser substitution at position 409 (N409S, also known as N370S) [29]. The H255Q and D409H variants are located in cis on the same allele, forming a complex allele (Fig. 4) [39, 40].

The heterozygous presence of the N370S allele and the H255Q+D409H complex allele defines a biallelic condition compatible with GD.

Subsequently, family segregation analysis showed that both parents (aged 59 and 53), who were clinically asymptomatic, had enzyme activity within the normal range (2.6 and 3.0 nmol/h/mL) (Table 4). Genetic analysis revealed the presence of the heterozygous H255Q and D409H variants in cis in the father, while the mother was a heterozygous carrier of the N370S variant, confirming that both were healthy carriers (Table 4).

The proband’s sister (aged 30), with a mild clinical presentation characterized by thrombocytopenia and mild splenomegaly (Table 4), showed a marked reduction in enzyme activity (0.4 nmol/h/mL) and an increase in LysoGb1 (257 ng/mL). Genetic analysis identified the same mutational profile as in the proband (N370S and the heterozygous H255Q+D409H allele complex), confirming the diagnosis of GD (Table 4) [39, 40]. This case emphasises the significance of family analysis in the context of hereditary genetic diseases, such as GD, where the clinical presentation may be atypical or mild. Currently, the proband and his sister are undergoing ERT.

**Table 4:** Clinical and molecular information on the proband of family 3 and his relatives. Values in bold are pathological

Patient	Sex	Age	Mutation	GCase Activity (nmol/mL/h) Normal Range > 2.5 nmol/mL/h	Lyso-Gb1 in Blood (ng/mL) Normal Range < 6.8 ng/mL	Clinical Signs
Proband	M	24	**N370S/H255Q+D409H**	**0.7**	**513**	Thrombocytopenia, hepatomegaly, and splenomegaly
Father	M	59	H255Q+D409H	2.6	//	Asymptomatic
Mother	F	53	N370S	3	//	Asymptomatic
Sister	F	30	**N370S/H255Q+D409H**	**0.4**	**257**	Thrombocytopenia and mild splenomegaly

### Clinical history family 4

The study of Family 4 was started following the presentation of a 30-year-old patient with a clinical suspicion of the disease, based on leucopenia and thrombocytopenia, who responded poorly to steroid therapy (Table 5). Performing the DBS test provided an initial biochemical profile, revealing values consistent with heterozygous carrier status, with acid β-glucosidase activity of 2 nmol/h/mL and LysoGb1 levels of 3.6 ng/mL (Table 5). Molecular analysis of the *GBA1* gene identified the single heterozygous variant c.955 A > C (Table 5). This variant, located in exon 7, results in the Met→Leu substitution (p.M319L; p.M280L after removal of the signal peptide). The extension of the study to the brother (aged 27), who presented with growth delay and a history of mild thrombocytopenia (approximately 130,000/µL) (Table 5), lead to the diagnosis of GD. Biochemical analysis revealed a marked reduction in enzymatic activity (0.1 nmol/h/mL) and an increase in LysoGb1 (10 ng/mL). Genetic analysis using Sanger Sequencing identified two compound heterozygous variants: c.955 A > C (p.M280L) [2] and the N370S mutation in exon 9, already widely associated with GD.

Following the diagnosis in the brother, a family linkage analysis was performed. The parents, who were clinically asymptomatic, were both found to be simple heterozygous carriers of a mutation (Table 5). The son of the affected individual (aged 2), who was asymptomatic, presented with a marked reduction in enzyme activity (0.3 nmol/h/mL) and an increase in LysoGb1 (14.3 ng/mL) (Table 5). Genetic analysis revealed the presence of the compound heterozygous variants p.M280L and N370S, confirming the diagnosis of GD (Table 5). The child’s mother (aged 30), who was clinically asymptomatic, had enzyme activity within normal limits (2.5 nmol/h/mL) and a LysoGb1 level of 8 ng/mL (Table 5). Genetic analysis identified the N370S mutation in heterozygous form, consistent with healthy carrier status (Table 5). The investigation was further extended to the maternal grandparents and uncle, all of whom were asymptomatic. Two individuals (the grandmother and uncle) were heterozygous carriers of a single variant, while the maternal grandfather had a wild-type genotype (Table 5).

The child’s younger sister (8 months), presenting with thrombocytopenia (86,000/µL), was also evaluated (Table 5). Biochemical analysis revealed a marked reduction in acid β-glucosidase activity (0.5 nmol/h/mL) and an increase in LysoGb1 (15.5 ng/mL). Genetic analysis confirmed the presence of the compound heterozygous variants p.M280L and N370S, allowing a diagnosis of GD in this case as well (Table 5). It is important to note that, in the absence of the ongoing family study, the sister’s neonatal diagnosis, given a non-specific presentation such as thrombocytopenia, would likely have been delayed or not considered.

At a current time, the father and his two affected sons are undergoing ERT.

**Table 5:** Clinical and molecular information on family 4. Values in bold are pathological

Patient	Sex	Age	Mutation	GCase Activity (nmol/mL/h) Normal Range > 2.5 nmol/mL/h	Lyso-Gb1 in Blood (ng/mL) Normal Range < 6.8 ng/mL	Clinical Signs
Paternal uncle	M	30	**M280L/N370S**	**2**	**3.6**	Leucopenia and thrombocytopenia
Father	M	27	**M280L/N370S**	**0.1**	**10**	Growth delay and thrombocytopenia
Mother	F	30	N370S	2.5	8	Asymptomatic
Daughter	F	8 month	**M280L/N370S**	**0.5**	**15.5**	Thrombocytopenia
Son	M	2	**M280L/N370S**	**0.3**	**14.3**	Asymptomatic
Paternal Grandfather	M	70	M280L	**1.4**	7.4	Asymptomatic
Paternal Grandmother	F	66	N370S	**1.4**	4.6	Asymptomatic
Maternal Grandfather	M	65	wt	3.2	7.4	Asymptomatic
Maternal Grandmother	F	56	N370S	2.8	9.8	Asymptomatic
Maternal uncle	M	38	N370S	**1.3**	//	Asymptomatic

## Discussion and conclusions

For a considerable years, our Research Centre has been actively engaged in the study of LSDs, with a particular focus on GD, a rare autosomal recessive genetic disorder caused by mutations in the *GBA1* gene that compromise the activity of the lysosomal enzyme GCase. As part of our diagnostic activities, we regularly receive biological samples from a large network of Italian specialist centres. These centres specialize in paediatrics, haematology, internal medicine and metabolic diseases, and together they contribute to the creation of a large and clinically heterogeneous database.

Our experience has highlighted a critical issue that is often underestimated in the management of GD: the lack of systematic application of family genetic studies, despite their decisive potential in improving early identification of the disease and correct characterization of the hereditary context. The absence of timely investigations in family members (especially non-direct relatives) of diagnosed patients can lead to significant delays in the diagnosis of other affected individuals or carriers. This, in turn, may hinder early and personalized care.

The present study was conducted with the aim of investigating this issue in depth, through the analysis of four distinct families. In the initial three cases, the investigation was initiated with a probands who had been diagnosed with GD, exhibiting a substantial decrease in GCase enzyme activity, pathological lysosomal accumulation, and the presence of two pathogenic variants of the *GBA1* gene. The investigation was extended to the family members of each proband through the combined use of enzymatic and molecular analyses. In all cases considered, following the diagnosis in the proband, family segregation studies were initially directed at first-degree blood relatives (parents and siblings) and then, where possible, extended to members of the extended family, such as grandparents, uncles, aunts and cousins. This methodological approach facilitated the precise identification of subjects who were either simple heterozygous carriers or those harbouring homozygous or compound heterozygous mutations, frequently in the presymptomatic phase or manifesting subtle clinical symptoms.

Of particular note was the fourth case analyzed, in which the initial identification of a heterozygous individual for a pathogenic variant was the starting point for a systematic extension of genetic testing to the entire family. This approach facilitated the identification of additional affected individuals, including newborns, underscoring the clinical and preventive value of family studies in GD, particularly in terms of early diagnosis and timely treatment. Specifically, the investigation did not begin with a first-degree relative, such as a parent or sibling, but with the paternal uncle. The latter was a simple heterozygous carrier of a pathogenic mutation, a condition which, in the absence of further clinical evidence, could reasonably have led to the conclusion of the investigation. However, subsequent attention was directed towards the uncle’s brother, who exhibited growth retardation, a clinical sign that is compatible with GD, as outlined in the literature [41]. Subsequent investigations, including genetic and enzymatic analyses, confirmed the initial diagnosis, thereby substantiating the presence of two compound heterozygous mutations and providing a definitive pathological characterization. In view of these findings, it was deemed essential to proceed with the expansion of family studies. This approach proved to be instrumental in identifying two additional affected individuals: the brother’s children, who were both clinically asymptomatic at the time of analysis. The case of the younger daughter, diagnosed in the neonatal phase (8 months of age), was of particular significance. This was due to the absence of clinical manifestations, which had previously prevented her from undergoing specific tests for GD. This emblematic case underscores the critical importance of expanded family studies, encompassing not only first-degree relatives, in the diagnosis and prevention of GD. The identification of carriers or affected individuals among more distant family members allows for the early detection of otherwise unrecognized clinical forms, thereby preventing the onset of irreversible damage to target organs and ensuring timely therapeutic intervention. This evidence serves to reinforce the objective of our work, which is to promote a proactive and inclusive diagnostic approach, capable of recognising the entire family dimension of the disease, thus overcoming the current tendency to underestimate the importance of testing non-direct relatives.

Despite the recognized importance of family segregation studies within the domain of medical genetics, these studies remain underexploited in clinical practice, particularly in the context of rare diseases. However, in the case of GD, family genetic tracking not only allows the transmission pattern of pathogenic variants to be accurately reconstructed, but is also essential for delineating the genetic and phenotypic context of the entire family unit [42, 43]. This is of particular pertinence in cases of attenuated phenotype, where diagnosis is hindered by the non-specificity of the clinical picture. Indeed, the clinical manifestation of GD can present with similarities to a range of haematological, rheumatological, or metabolic conditions. Symptoms such as splenomegaly, thrombocytopenia, bone pain or asthenia, which are often non-specific, have been shown to delay diagnosis or lead to diagnostic errors, as documented by numerous studies [4, 13, 44]. In such cases, family investigation is a strategic tool for identifying undiagnosed forms, especially in paediatrics or in individuals with insidious onset.

Consequently, molecular analysis of the *GBA1* gene is confirmed as a diagnostic resource of primary importance. The functionality in question permits: 1), the diagnosis must be confirmed in probands; 2); the genetic status of family members must be determined (whether they are affected, carriers or healthy); 3) guidance must be provided on personalized clinical and therapeutic pathways. 4) genetic counselling, surveillance and reproductive planning must be guided. Early identification of affected individuals also allows for the timely initiation of specific therapies, such as ERT or pharmacological chaperones, with the aim of slowing disease progression and preventing irreversible organ damage.

In view of the findings, we propose the systematic incorporation of family studies into the GD diagnostic pathway. This approach must not be regarded as an optional addition to clinical management; rather, it should be considered an integral part of the overall strategy. The findings of this study demonstrate that family screening can lead to substantial improvements in clinical outcomes through the early, accurate and multidimensional identification of the disease. The findings of this study have facilitated the implementation of personalized clinical monitoring strategies and the timely initiation of targeted treatments for affected individuals, contributing to the attenuation of the progression of the disease and the substantial enhancement of clinical outcomes.

## Data Availability

Data are contained within the article.
